# Thirty-six months recurrence after acute ischemic stroke among patients with comorbid type 2 diabetes: A nested case-control study

**DOI:** 10.3389/fnagi.2022.999568

**Published:** 2022-09-30

**Authors:** Lu Wang, Hongyun Li, Jiheng Hao, Chao Liu, Jiyue Wang, Jingjun Feng, Zheng Guo, Yulu Zheng, Yanbo Zhang, Hongxiang Li, Liyong Zhang, Haifeng Hou

**Affiliations:** ^1^School of Public Health, Shandong First Medical University and Shandong Academy of Medical Sciences, Taian, China; ^2^Department of Neurosurgery, Liaocheng People’s Hospital, Liaocheng, China; ^3^School of Medical and Health Sciences, Edith Cowan University, Joondalup, WA, Australia; ^4^The Second Affiliated Hospital of Shandong First Medical University, Taian, China

**Keywords:** ischemic stroke, diabetes mellitus, recurrence, risk factors, nested case-control study

## Abstract

**Background:**

Stroke patients have to face a high risk of recurrence, especially for those with comorbid T2DM, which usually lead to much more serious neurologic damage and an increased likelihood of death. This study aimed to explore determinants of stroke relapse among patients with comorbid T2DM.

**Materials and methods:**

We conducted this case-control study nested a prospective cohort of ischemic stroke (IS) with comorbid T2DM. During 36-month follow-up, the second stroke occurred in 84 diabetic IS patients who were allocated into the case group, while 613 patients without recurrence were the controls. We collected the demographic data, behaviors and habits, therapies, and family history at baseline, and measured the variables during follow-up. LASSO and Logistic regression analyses were carried out to develop a prediction model of stroke recurrence. The receiver operator characteristic (ROC) curve was employed to evaluate the performance of the prediction model.

**Results:**

Compared to participants without recurrence, the higher levels of pulse rate (78.29 ± 12.79 vs. 74.88 ± 10.93) and hypertension (72.6 vs. 61.2%) were recorded at baseline. Moreover, a lower level of physical activity (77.4 vs. 90.4%), as well as a higher proportion of hypoglycemic therapy (36.9 vs. 23.3%) was also observed during 36-month follow-up. Multivariate logistic regression revealed that higher pulse rate at admission (OR = 1.027, 95 %CI = 1.005–1.049), lacking physical activity (OR = 2.838, 95% CI = 1.418–5.620) and not receiving hypoglycemic therapy (OR = 1.697, 95% CI = 1.013–2.843) during follow-up increased the risk of stroke recurrence. We developed a prediction model using baseline pulse rate, hypoglycemic therapy, and physical activity, which produced an area under ROC curve (AUC) of 0.689.

**Conclusion:**

Physical activity and hypoglycemic therapy play a protective role for IS patients with comorbid diabetes. In addition to targeted therapeutics, the improvement of daily-life habit contributes to slowing the progress of the IS.

## Background

Globally, stroke has ranked as the second leading cause of death (Wu Y. et al., 2019; Zheng et al., 2022) and the third leading cause of disability (Cao et al., 2019; Collaborators, 2021). Ischemic stroke (IS), characterized by temporary or permanent cerebrovascular occlusion (Liu et al., 2018), accounts for 82% of acute stroke events (Mehra et al., 2019; Feske, 2021). The incidence and mortality of stroke are significantly higher in China than in developed countries: a study on the Global Burden of Disease in 2019 reported stroke as the leading cause of death in this country (Wu S. et al., 2019).

Stroke patients face a high likelihood of recurrence after the initial onset of IS, ranging from 5.4% at 1 year to 11.3% at 5 years (Khanevski et al., 2019). Patients with stroke recurrence have a higher likelihood to endure more serious brain damage and neurologic dysfunction, leading to a worse functional status and/or higher case fatality rate vs. initial stroke (Lin B. et al., 2021; Wang et al., 2021). Physical activity, antihypertensive treatment, and high fiber intake (i.e., fruits and vegetables) have preventive effects on IS recurrence (Gillman et al., 1995; Zonneveld et al., 2018; Lin Y. et al., 2021). Other modifiable lifestyle characteristics, including obesity, hypertension, diabetes, depression and smoking, usually serve as risk factors (Black et al., 2015; Chen et al., 2019; Huang et al., 2019; Zheng and Yao, 2019; Cao et al., 2020; Szlachetka et al., 2020; Kumral et al., 2021). Despite recent progress in the management and prevention of recurrent stroke, enhanced approaches are needed to further reduce relapse risk.

Diabetes mellitus (DM), a complex disease featuring a deficiency or resistance to insulin, exposes individuals to hyperglycemia (Tang et al., 2014). This state causes pathologic changes in the blood vessels of various organs, including cerebral vessels (Li et al., 2020). The incidence of cardiovascular diseases, such as IS, is apparently higher in patients with DM than in persons without. As one of established risk factors for stroke, the prevalence of DM-induced IS has been increasing in all age groups (Benjamin et al., 2019; Venkat et al., 2021). Acute stroke with comorbid DM has significantly higher risks of aggravated pathology, disability, and death. These consequences mostly arise from extensive unbalance of metabolism, severe vascular damage, deteriorated white matter, and specific inflammatory milieu (Ergul et al., 2016; Venkat et al., 2017, 2021). As understood, diabetic stroke patients face a higher probability of stroke recurrence. Controlling glucose levels and other risk factors has been considered an effective means of preventing subsequent strokes (Lau et al., 2019). However, few studies have focused on precise prevention strategies for stroke recurrence in Chinese patients with comorbid DM. Identifying determinants of stroke recurrence in DM patients will help to alleviate stroke-related deterioration. Findings will also advance personalized healthcare strategies.

## Materials and methods

### Study participants

This nested case–control study was conducted with a Chinese Han stroke cohort established at the Brain Hospital of Liaocheng People’s Hospital (BHLPH) in Shandong Province, China. This cohort enrolled 1,793 patients with acute IS who were admitted to BHLPH between January 1, 2017, and September 30, 2018. Among these patients, 717 had T2DM. After 36 months of follow-up, patients with recurrent stroke were recruited in the case group; those without recurrence constituted the control group.

Study inclusion criteria were as follows: (1) First-ever stroke; (2) with comorbid T2DM; (3) a diagnosis of acute IS, confirmed via magnetic resonance imaging with reference to the Chinese Guidelines for the Diagnosis and Treatment of Acute Ischemic Stroke (Neurology and Society, 2018); (4) age ≥ 18 years; and (5) complete clinical data were available. The exclusion criteria covered (1) patients with severe mental disorders; (2) pregnant or breastfeeding women; (3) patients with incomplete clinical characteristics; (4) patients with severe somatic diseases (e.g., heart diseases, cancers, and infectious diseases); and (5) patients enrolled in other clinical trials.

The study protocol was reviewed and approved by the ethics committee of BHLPH (No. BHLPH085). All participants provided their written informed consent.

### Data collection

Participants’ demographic data were obtained through face-to-face interviews. Clinical features were collected by trained physicians. According to body mass index (BMI), the participants were classified were classified as (1) underweight (BMI < 18.5), (2) normal weight (BMI of 18.5–23.9), (3) overweight (BMI of 24.0–27.9), or (4) obese (BMI ≥ 28). The National Institutes of Health Stroke Scale (NIHSS), a 15-item neurologic-impairment scale with scores ranging from 0 (no deficit) to 42 (quadriplegia and coma) was used to assess acute stroke deficits. NIHSS frequencies were classified into 5 groups: no measured stroke symptoms (0); minor stroke (1–4); moderate stroke (5–15); moderate to severe stroke (16–20); and severe stroke (21–42) (Saber and Saver, 2020). The Modified Rankin Scale (mRS) is a disability scale ranging from 0 (no symptoms) to 6 (death), by which patients were classified into two groups: good outcome (0–2), and worse outcome (3–6) (Armangue et al., 2018). A fasting venous blood sample was collected for determination of hematologic and biochemical parameters, including high-density lipoprotein cholesterol (HDL-C), low-density lipoprotein cholesterol (LDL-C), triglycerides (TG), total cholesterol (TC), fasting blood glucose (FBG), and total homocysteine (tHcy), were quantified with an automatic analyzer (Hitachi, Tokyo, Japan). Pulse rate was automatically recorded by a finger pulse oximetric device (Yuwell, Jiangsu, China). Blood pressure (BP) was measured by a trained nurse in a sitting position with at least 5 min rest before the first measurement, and the mean of two measurements was recorded.

The 36-month follow-up was performed by a trained clinical neurologist after patients were discharged from the hospital. Parameters including stroke recurrence, age, gender, smoking, alcohol use, systolic blood pressure (SBP), diastolic blood pressure (DBP), FBG, physical activity (often: ≥ 3 sessions per week and ≥ 30 min per session, or moderate-intensity exercise), family history, and medications were obtained when visited annually. The mean of each variable was calculated during the follow-up period.

Patients who take the medicines as prescribed are regarded as receiving the relevant treatment: (1) Antiplatelet therapy (e.g., aspirin, clopidogrel, or ticagrelor), (2) antihypertensive therapy [e.g., angiotensin-converting enzyme inhibitors (ACEI), angiotensin receptor blocker (ARB)], (3) hypoglycemic therapy (e.g., insulin, pioglitazone), (4) antithrombotic therapy (e.g., aspirin, ozagrel), and (5) lipid-lowering drug therapy (e.g., statins, niacin and its derivatives).

### Statistical analysis

For the purposes of reporting results, continuous variables are expressed herein as means and standard deviations; categorical variables are presented as frequencies and/or percentages. The Kolmogorov–Smirnov test was used to examine the normal distribution of continuous data. If normality was assumed, a Student’s *t*-test was performed to analyze the difference between groups; otherwise, the data were analyzed using a Mann–Whitney *U*-test. Meanwhile, between-group differences in categorical data were tested via a chi-square test. To alleviate collinearity between multiple variables, a least absolute shrinkage and selection operator (LASSO) regression was carried out to select variables potentially associated with IS recurrence in patients with comorbid T2DM. A multivariate logistic regression was performed to screen statistically significant variables: The odds ratio (OR) and 95% confidence interval (CI) were calculated for each variable. Then, a multivariate prediction model was developed using these significant variables. To evaluate the model’s performance in predicting stroke recurrence, a receiver operator characteristic (ROC) curve was constructed, and the area under the ROC curve (AUC) was determined. In addition, a nomogram was designed on the basis of the multivariate logistic regression model; the concordance index (C-index) was used to assess the nomogram’s prediction accuracy.

Statistical analyses were performed in SPSS 26.0 (IBM, NY) and R packages 4.1.0 (R Core Team). A two-tailed *P*-value of less than 0.05 was considered statistically significant.

## Results

### Characteristics of study participants

**FIGURE 1:**
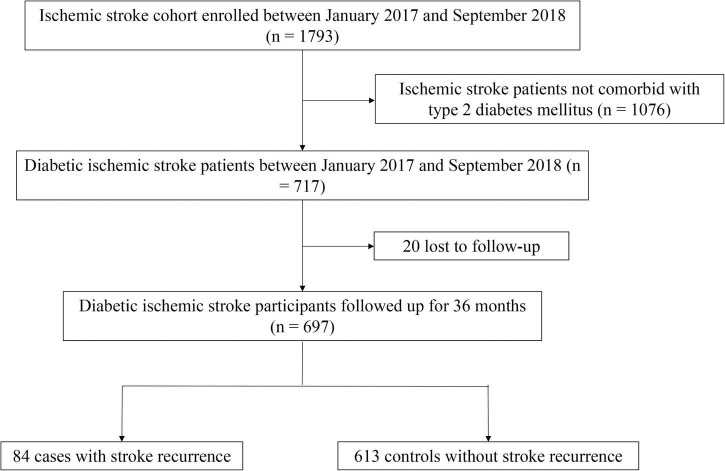
Flow chart for study participant selection.

Twenty individuals were lost to follow-up after 36 months (2.8%, 20/717). Stroke recurred in 84 (12.05%) of participants in the case group, while 613 patients without recurrence were allocated into the control group. Study participants were aged 65.2 ± 11.28 years, of whom 61.4% (428/697) were men. Figure 1 presents a flowchart describing study population selection.

**FIGURE 2:**
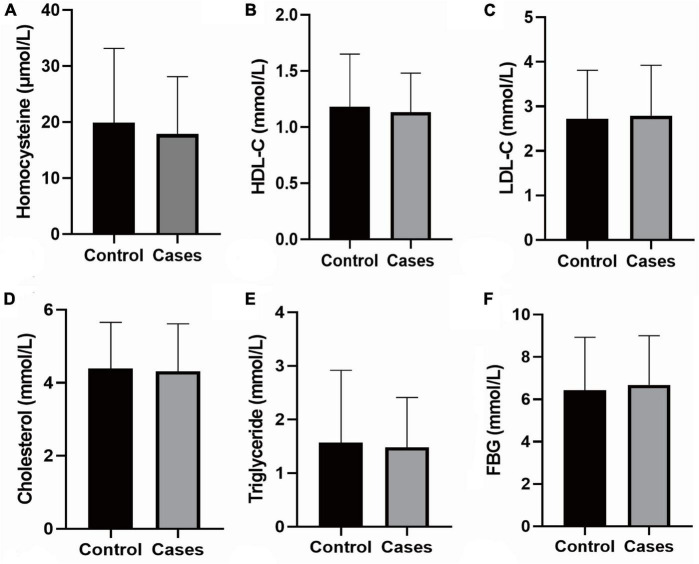
Biochemical tests. **(A)** The level of Homocysteine; **(B)** the level of HDL-C; **(C)** the level of LDL-Cs; **(D)** the level of Cholesterol; **(E)** the level of Triglyceride; **(F)** the level of FBG; the data shown in the graphs represent the mean ± SD. FBG, fasting blood-glucose; LDL-C, low density lipoprotein cholesterol; HDL-C, high-density lipoprotein cholesterol; SD, standard deviations.

Participants’ baseline data are summarized in Table 1 and Supplementary Table 1. Differences in age, gender, BMI, smoking, alcohol consumption, FBG, triglycerides, TC, LDL-C, HDL-C, tHcy, neck vascular stenosis, Family history of stroke, coronary heart disease and hypertension, mRS score on admission, and NIHSS score on admission were not statistically significant between the case and control groups. Patients with stroke recurrence had a higher prevalence of hypertension at admission than the control group (*P* < 0.05). In addition, the pulse rate of recurrent cases was significantly higher than the controls (*P* < 0.05). The results of biochemical tests are shown in Figure 2.

**TABLE 1:** Baseline data of participants.

Variables	Cases of recurrence (*N* = 84)	Cases of non-recurrence (*N* = 613)	t/χ^2^	*P*
**Age group (*n*, %)**				
< 60	23 (27.4)	197 (32.1)	1.493	0.474
60−	25 (29.8)	194 (31.6)		
≥ 70	36 (42.9)	222 (36.2)		
**Gender (*n*, %)**				
Male	52 (61.9)	376 (61.3)	0.010	0.920
Female	32 (38.1)	237 (38.7)		
**BMI classification (*n*, %)**				
Normal (18.5–23.9)	34 (40.5)	232 (37.8)	0.370	0.831
Overweight (24–27.9)	38 (45.2)	299 (48.8)		
Obese (≥ 28)	12 (14.3)	82 (13.4)		
**Smoking (*n*, %)**				
Yes	69 (82.1)	463 (75.5)	1.788	0.181
No	15 (17.9)	150 (24.5)		
**Alcohol consumption (*n*, %)**				
Yes	62 (73.8)	430 (70.1)	0.477	0.490
No	22 (26.2)	183 (29.9)		
**Hypertension (*n*, %)**				
Yes	61 (72.6)	375 (61.2)	4.131	0.042
No	23 (27.4)	238 (38.8)		
**Neck vascular stenosis (*n*, %)**				
Yes	20 (23.8)	205 (33.4)	3.136	0.077
No	64 (76.2)	408 (66.6)		
**Family history of stroke (*n*, %)**				
Yes	3 (3.6)	59 (9.6)	3.340	0.068
No	81 (96.4)	554 (90.4)		
**Family history of CHD (*n*, %)**				
Yes	1 (1.2)	29 (4.7)	1.471	0.225
No	83 (98.8)	584 (95.3)		
**Family history of hypertension (*n*, %)**				
Yes	5 (6.0)	45 (7.3)	0.214	0.644
No	79 (94.0)	568 (92.7)		
**mRS score at admission (*n*, %)**				
Low (0–2)	497 (81.1)	72 (85.7)	1.060	0.303
High (3–5)	116 (18.9)	12 (14.3)		
**NIHSS score on admission (*n*, %)**				
Normal (0)	20 (23.8)	128 (20.9)	3.932	0.415
Minor stroke (1–4)	26 (31.0)	186 (30.3)		
Moderate stroke (5–15)	22 (26.2)	132 (21.5)		
Moderate to severe stroke (16–20)	16 (19.0)	13 (2.1)		
Severe stroke (21–42)	0 (0.0)	154 (25.1)		
**Pulse rate (times/min)**	78.29 ± 12.79	74.88 ± 10.93	2.619	0.009

BMI, body mass index; CHD, coronary heart disease; NIHSS, National Institutes of Health Stroke Scale; mRS, Modified Rankin Scale.

### Follow-up

As shown in Table 2 and Supplementary Table 1, no significant between-group differences were detected for variables including smoking; FBG; mRS score, rehabilitation treatment; and antihypertension, antiplatelet, antithrombosis, and lipid-lowering therapies. The proportions of physical activity and hypoglycemic therapy were statistically different between groups (*P* < 0.05).

**TABLE 2:** Parameters during follow-up.

Characteristics	Cases of recurrence (*N* = 84)	Cases of non-recurrence (*N* = 613)	χ^2^	*P*
**Smoking (*n*, %)**				
Yes	74 (88.1)	513 (83.7)	1.08	0.299
No	10 (11.9)	100 (16.3)		
**Physical activity (*n*, %)**				
Often*	65 (77.4)	554 (90.4)	12.551	< 0.001
Lacking	19 (22.6)	59 (9.6)		
**mRS score (*n*, %)**				
Low (0–2)	76 (90.5)	568 (92.7)	0.501	0.479
High (3–5)	8 (9.5)	45 (7.3)		
**FBG**				
<7.0 mmol/L	75 (89.3)	572 (93.3)	1.798	0.180
≥7.1 mmol/L	9 (10.7)	41 (6.7)		
**Antiplatelet therapy (*n*, %)**				
Yes	65 (77.4)	487 (79.4)	0.191	0.662
No	19 (22.6)	126 (20.6)		
**Antihypertensive therapy (*n*, %)**				
Yes	46 (54.8)	312 (50.9)	0.442	0.506
No	38 (45.2)	301 (49.1)		
**Hypoglycemic therapy (*n*, %)**				
Yes	31 (36.9)	143 (23.3)	7.270	0.007
No	53 (63.1)	470 (76.7)		
**Rehabilitation therapy (*n*, %)**				
Yes	58 (69.0)	444 (72.4)	0.420	0.517
No	26 (31.0)	169 (27.6)		
**Antithrombotic therapy (*n*, %)**				
Yes	45 (53.6)	317 (48.3)	0.102	0.749
No	39 (46.4)	296 (51.7)		
**Lipid-lowering drug (*n*, %)**				
Yes	48 (57.1)	298 (48.6)	2.150	0.143
No	36 (42.9)	315 (51.4)		

mRS, Modified Rankin Scale; FBG, fasting blood glucose.

* ≥ 3 sessions per week and ≥ 30 min per session, or moderate-intensity exercise.

### Development of prediction model of stroke recurrence

**FIGURE 3:**
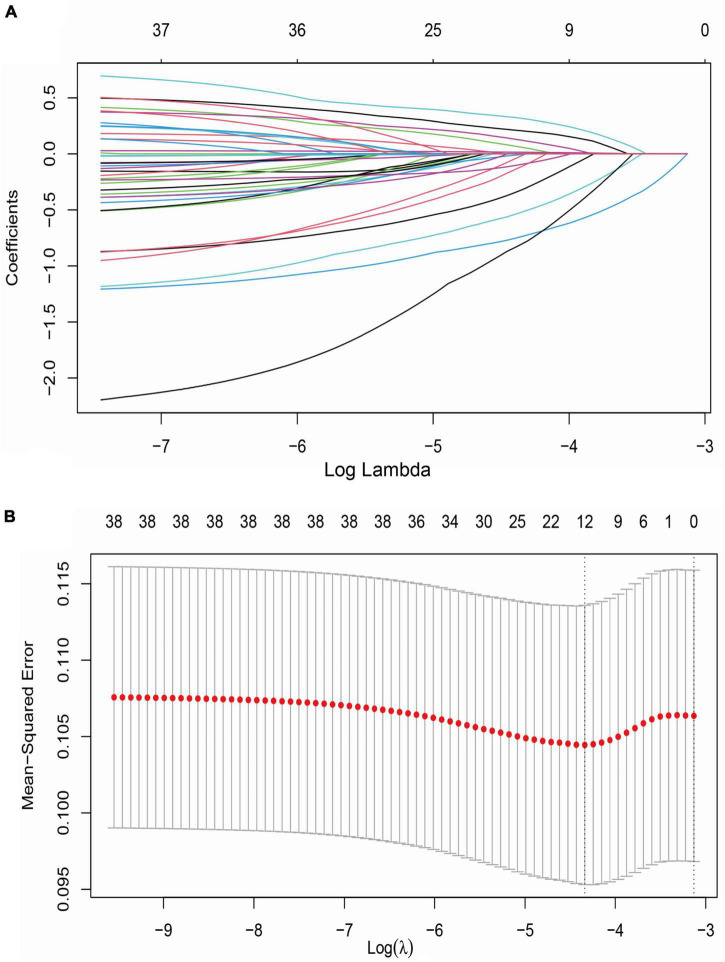
LASSO regression analysis for variable selection. **(A)** LASSO coefficient of 38 variables; **(B)** optimal penalty coefficient (λ = 0.000584) in LASSO regression identified with the minimum criterion; LASSO, least absolute shrinkage and selection operator; SE, standard error.

A LASSO model was used to screen potential determinants associated with stroke recurrence in T2DM patients. In Figure 3, red dots denote the target parameter to which each λ corresponds; the two dotted lines refer to two special λ values. Each curve matched the track of a single covariate coefficient. Finally, 38 covariates were selected for this model, with an optimal λ of 0.000584.

**FIGURE 4:**
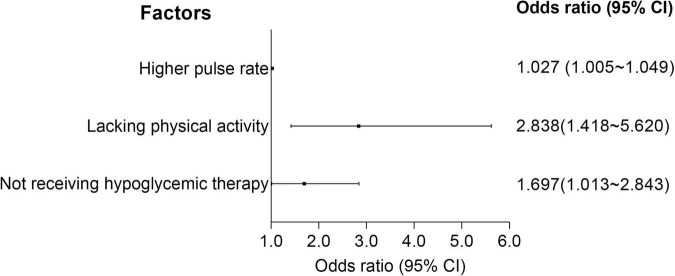
Forest plots of logistic regression. CI, confidence interval.

A logistic regression model, along with the LASSO regression, was established to screen the determinants of stroke recurrence. After adjusting for age, sex, BMI, tHcy, LDL-C, HDL-C, cholesterol, smoking and family history of stroke, a higher pulse rate at admission (OR = 1.027, 95% CI = 1.005–1.049) was significantly associated with increased risk for recurrence of stroke. Not receiving Hypoglycemic therapy (OR = 1.697, 95% CI = 1.013–2.843) and lacking physical activity during follow-up (OR = 2.838, 95% CI = 1.418–5.620) correlated with a higher risk for recurrence (Figure 4 and Supplementary Table 2).

### Performance of prediction model

**FIGURE 5:**
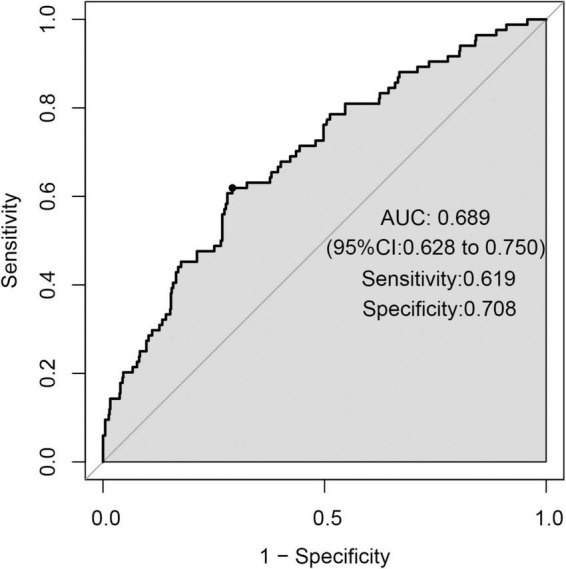
Receiver operator characteristic (ROC) curve.

The ROC curve analysis (Figure 5) showed a high performance of differentiation between high and low risk for stroke recurrence (AUC = 0.689, 95% CI = 0.628–0.750). According to the ROC curve, our prediction model exerted a relatively high accuracy, with a sensitivity of 61.9% and a specificity of 70.8% at a cut-off point of 0.130.

**FIGURE 6:**
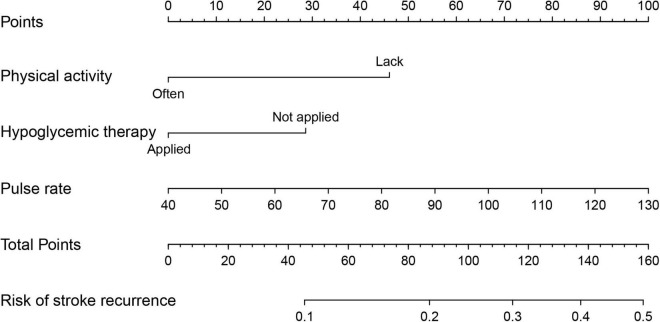
Nomogram to predict 36-month risk of stroke recurrence. Draw a line perpendicular from the corresponding axis of each factor until it reaches the top line labeled “Points”. Sum up the number of points for all factors, then draw a line descending from the axis labeled “Total Points” until it intercepts each of the axes to predict 36-month risk of stroke recurrence.

A nomogram was constructed with the above three predictors to forecast the 36-month risk of stroke recurrence (Figure 6). Internal validation was based on the random 70:30 partitioning of study participants data into training data: Test data sets. The nomogram’s prediction accuracy was evaluated by the C-index: 0.624 (95% CI = 0.553–0.695) for the training set and 0.653 (95% CI = 0.513–0.793) for the test set. As such, the nomogram displayed relatively good performance.

## Discussion

The recurrence rate of stroke was 12.05% over 36 months of follow-up in this diabetic IS cohort. Higher pulse rate at admission, physical inactivity and not receiving hypoglycemic therapy during follow-up increased the risk of stroke recurrence among patients with comorbid T2DM. Our prediction model based on the three risk factors appeared capable of identifying recurrence risk.

Accurately identifying modifiable risk factors for stroke recurrence is crucial for developing strategies to lower stroke-related mortality and morbidity. Compared with first-ever stroke, patients with recurrence have markedly more severe functional disability and higher mortality (Andreasen et al., 2018). One randomized controlled trial found that DM is associated with a higher risk of new stroke in patients with minor stroke (Chen et al., 2017). Although prevention measures targeting stroke have lessened relapse rates, patients with recurrence account for nearly 30% of all stroke cases (He et al., 2020). Several determinants correlated with stroke recurrence have been acknowledged (Kolmos et al., 2021). However, few studies have been conducted on IS with comorbid T2DM in China. Within the Chinese Han population profiled in this research, we observed 12.05% stroke recurrence during 36 months of follow-up, consistent with previous studies (Andersen et al., 2015; He et al., 2015; Khanevski et al., 2019).

IS patients with comorbid T2DM have poorer clinical outcomes and long-term prognoses than those with normal blood glucose levels (Kang and Park, 2017). Hyperglycemia increases stroke patients’ risk of death by 87% at 30 days, 75% at 1 year, and 41% at 6 years after stroke, respectively (Johnston and Beckman, 2019). In addition to being an independent predictor of stroke-induced death (Geary et al., 2019), hyperglycemia is a determinant of stroke recurrence (Hotter et al., 2019). Hyperglycemia augments brain injury through multiple potential mechanisms, including endothelial dysfunction, oxidative stress, increment of inflammatory response, and impaired fibrinolysis (Garg et al., 2006; MacDougall and Muir, 2011; Nogueira-Machado et al., 2011; Krinock and Singhal, 2021). Compared with patients who have normal blood glucose levels, hyperglycemia leads to a 2.5-fold increase in the risk of 90-day stroke recurrence (Guo et al., 2021). Our between-group comparisons revealed the proportion of hypoglycemic therapy to be lower in patients with recurrent stroke. Multivariate logistic regression contrarily demonstrated that not receiving hypoglycemic therapy was associated with a higher risk of recurrence after adjusting for age, sex, BMI, tHcy, LDL-C, HDL-C, cholesterol, family history, smoking, and pulse rate. This finding corroborates clinical trials and a systematic review (Lee et al., 2017; Spence et al., 2019). Hypoglycemic agents such as pioglitazone reduce the risk of stroke recurrence by activating PPARγ signaling in adipocytes, immune and endothelial cells, and other tissues (Kernan et al., 2016; Yaghi et al., 2018). This process contributes to a lower likelihood of stroke recurrence among patients with insulin resistance, prediabetes, or DM (Yang et al., 2018).

Physical activity can lower venous pressure, elevate venous flow, and reduce thrombosis (Johansson et al., 2019). Physical activity also helps reduce the risk of atherosclerosis by boosting lipid metabolism and raising blood levels of HDL-C (Sun et al., 2021). These effects can prevent relapse in atherosclerotic stroke patients (Waters et al., 2016; Sun et al., 2018; Gardener et al., 2019). Clinical studies have documented that physical activity and exercise enhance neuroplasticity and change brain activity patterns in post-stroke survivors, reducing the risk of recurrence (Kramer et al., 2019). A dose–response relationship has been identified between physical activity duration and stroke recurrence as well (Hou et al., 2021). Regular exercise results in genome-wide epigenetic modifications in human skeletal muscle and adipose tissue, which could affect metabolic phenotypes associated with stroke (Ling and Rönn, 2014). Our study revealed a significant relationship between physical inactivity and stroke relapse, similar to relevant studies (Oza et al., 2017; Turan et al., 2017).

In terms of pulse waves, scholars have taken pulse wave velocity and heart rate as major parameters when assessing arteriosclerosis development (Munakata, 2014; Townsend et al., 2015; Ueki et al., 2017). Increased pulse pressure may expose cerebral small vessels to high pulsatile pressure and flow, which damage the cerebral microvasculature and hinder the restoration of stroke-impaired neurological function (O’Rourke and Safar, 2005; Ishizuka et al., 2016). Pulse rate has been proposed as a surrogate for heart rate (Chang and Chang, 2009). Although the direct connection between stroke and pulse rate has yet to be clearly explained, epidemiological evidence implies that an elevated resting heart rate is associated with cardiovascular morbidity and mortality (Hjalmarson et al., 1990). Experimental and clinical findings suggest that sustained heart rate elevation, independent of the underlying trigger, is associated with the pathogenesis of cardiovascular diseases; pharmacological or interventional heart rate reduction can help to mitigate cardiovascular outcomes (Custodis et al., 2010). Animal studies have illustrated that a high heart rate contributes to upregulation of vascular oxidative stress, endothelial dysfunction, and accelerated atherogenesis (Custodis et al., 2008). Clinical studies point to an association between increased resting heart rate and inflammatory markers (i.e., C-reactive protein [CRP], white blood cell count, and fibrinogen) (Sajadieh et al., 2004; Rogowski et al., 2007). In this study, we illustrated that pulse rate is associated with increased risk of stroke recurrence.

Several limitations of our research require attention. First, this study was conducted with a single cohort from one medical center in northern China. This factor might cause Berkson’s bias, limiting the generalizability of findings. Second, because this study entailed a long-term follow-up among stroke patients, loss to follow-up might underestimate recurrence. Third, potentially significant variables may have been overlooked at baseline due to this study’s limited aims. Information bias could arise as a result.

## Conclusion

Our findings indicate that higher pulse rate at admission, physical inactivity and not receiving hypoglycemic therapy during follow-up expose the individuals with comorbid T2DM to higher risk of stroke recurrence. This result underlines the importance of healthy lifestyle behaviors and targeted therapeutics in alleviating the adverse outcomes of stroke.

## Data availability statement

The data analyzed in this study is subject to the following licenses/restrictions: All data and methods supporting the findings of this study are available from the corresponding author upon reasonable request. Requests to access these datasets should be directed to YBZ, bbnnbn@163.com.

## Ethics statement

The studies involving human participants were reviewed and approved by the Ethics Committee of BHLPH (No. BHLPH085). The participants provided their written informed consent to participate in this study.

## Author contributions

HH, HXL, LZ, and YBZ designed the study and took responsibility for the integrity of the data and the accuracy of the data analysis. LW, HYL, JH, CL, JW, JF, ZG, and YLZ contributed to data collection. LW, HYL, and JH contributed to statistical analysis and manuscript writing. HXL, LZ, and YBZ revised the manuscript. All authors made a significant intellectual contribution and approved the final version.
