# Supplementary material for: Thirty-six months recurrence after acute ischemic stroke among patients with comorbid type 2 diabetes: A nested case-control study
Source: Front Aging Neurosci. 2022 Sep 30;14:999568. doi: 10.3389/fnagi.2022.999568 (PMC9562049; doi:10.3389/fnagi.2022.999568)
Supplement: Supplementary file 1 [file Table_1.DOCX]

**Table S1** Fifteen variables at baseline and during follow-up

| **Variables** | **Cases of recurrence**  **(*N* = 84)** | **Cases of non-recurrence**  **(*N* = 613)** | ***t/*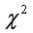** | ***P*** |
| --- | --- | --- | --- | --- |
| **FBG (mmol/L)** | 78.29±12.79 | 74.88±10.93 | 2.619 | 0.009 |
| **Triglyceride (mmol/L)** | 6.68±2.33 | 6.43±2.50 | 0.862 | 0.389 |
| **Cholesterol (mmol/L)** | 1.48±0.93 | 1.57±1.35 | 0.592 | 0.554 |
| **LDL-C (mmol/L)** | 4.31±1.30 | 4.40±1.26 | 0.584 | 0.560 |
| **HDL-C (mmol/L)** | 2.79±1.13 | 2.72±1.09 | 0.536 | 0.592 |
| **Homocysteine (μmol/L)** | 1.13±0.35 | 1.18±0.47 | 0.942 | 0.347 |
| **Blood pressure (n,%)** |  |  |  |  |
| Normal | 19 (22.6) | 158 (25.8) | 0.388 | 0.533 |
| Abnormal | 65 (77.4) | 455 (74.2) |  |  |
| **NIHSS score at discharge (n,%)** |  |  |  |  |
| Normal (0) | 43 (51.2) | 323 (52.7) | 0.983 | 0.885 |
| Minor stroke (1–4) | 28 (33.3) | 205 (33.4) |  |  |
| Moderate stroke (5–15) | 13 (15.5) | 75 (12.2) |  |  |
| Moderate to severe stroke (16-20) | 0 (0) | 6 (1) |  |  |
| Severe stroke (21-42) | 0 (0) | 4 (0.7) |  |  |
| **Dyslipidemia (n,%)** |  |  |  |  |
| Yes | 54 (64.3) | 374 (61.0) | 0.334 | 0.563 |
| No | 30 (35.7) | 239 (39.0) |  |  |
| **Blood sugar (n,%)** |  |  |  |  |
| Normal | 75 (89.3) | 571 (93.1) | 1.625 | 0.202 |
| Abnormal | 9 (10.7) | 42 (6.9) |  |  |
| **Hypoglycemic therapy during hospitalization (n, %)** |  |  |  |  |
| Yes | 26 (31.0) | 135 (22.0) | 3.316 | 0.069 |
| No | 58 (69.0) | 478 (78.0) |  |  |
| **Alcohol consumption during follow-up (n, %)** |  |  |  |  |
| Yes | 5 (6.0) | 103 (16.8) | 0.477 | 0.490 |
| No | 79 (94.0) | 510 (83.2) |  |  |
| **anticoagulant therapy (n, %)** |  |  |  |  |
| Yes | 2 (2.4) | 34 (5.5) | 1.511 | 0.219 |
| No | 82 (97.6) | 579 (94.5) |  |  |

FBG, fasting blood glucose; LDL-C, low-density lipoprotein cholesterol; HDL-C, high-density lipoprotein cholesterol; NIHSS, National Institutes of Health Stroke Scale
