# Supplementary material for: Thirty-six months recurrence after acute ischemic stroke among patients with comorbid type 2 diabetes: A nested case-control study
Source: Front Aging Neurosci. 2022 Sep 30;14:999568. doi: 10.3389/fnagi.2022.999568 (PMC9562049; doi:10.3389/fnagi.2022.999568)
Supplement: Supplementary file 2 [file Table_2.DOCX]

**Table S2** Logistic regression analysis for recurrence of stroke

| **Variables** | ***β*** | ***SE*** | **Walds χ^2^** | ***P*** | **OR (95%CI)** |
| --- | --- | --- | --- | --- | --- |
| Higher pulse rate | 0.026 | 0.011 | 5.820 | 0.016 | 1.027 (1.005~1.049) |
| Lacking physical activity during follow-up | 1.038 | 0.351 | 8.728 | 0.003 | 2.838(1.418~5.620) |
| Not receiving hypoglycemic therapy during follow-up | 0.529 | 0.263 | 4.037 | 0.045 | 1.697(1.013~2.843) |

SE, standard error; OR, odds ratio; CI, confidence intervals; *β*, regression coefficient
